# Can Telemedicine Support the Management of Osteogenesis Imperfecta?

**DOI:** 10.1590/1413-785220263401e295240

**Published:** 2026-03-30

**Authors:** Paulo Humberto Mem Martendal Vallandro Costa, Fernando Henrique Vilella Nakamuta, Vivian Yumi Aoki Nishida, Ellen de Oliveira Goiano, Miguel Akkari, Cláudio Santili

**Affiliations:** 1 Santa Casa de Misericordia de Sao Paulo, Pavilhao Fernandinho Simonsen, Departamento de Ortopedia e Traumatologia, Sao Paulo, SP, Brazil.; 2 Universidade de Sao Paulo, Escola Politecnica, Departamento de Engenharia de Producao, Sao Paulo, SP, Brazil.; 3 Santa Casa de Misericordia de Sao Paulo, Departamento de Fisioterapia, Sao Paulo, SP, Brazil.

**Keywords:** Telemedicine, Osteogenesis Imperfecta, Rehabilitation, Physical Therapy, Health Technology, Telemedicina, Osteogênese Imperfeita, Reabilitação, Fisioterapia, Tecnologia em Saúde

## Abstract

**Objective:**

Considering the need for specialized care in Osteogenesis Imperfecta (OI), we investigated whether the use of a digital platform could optimize treatment and improve patients’ quality of life.

**Methods:**

A guidance and data collection app was developed, complemented by a YouTube channel with physiotherapy instructions. The interventional clinical trial without a control group was carried out for 12 months with patients with OI. Patient functionality was assessed using the Functional Independence Measure (FIM) scale. Patients were monitored via telemedicine, receiving remote multidisciplinary support.

**Results:**

Thirteen participants completed the study. Significant improvements were observed in self-care, transfers, and mobility subitems (p<0.05). Program adherence was high, and no patient sustained injuries during the intervention. Additionally, a total transportation cost savings of R$ 113,852.00 was recorded, reducing direct expenses for patients and their families.

**Conclusion:**

Telemedicine proved effective in the rehabilitation of OI patients, providing both clinical and financial benefits. The digital platform facilitated treatment adherence, optimizing medical and physiotherapy support. Future studies should explore its large-scale application and integration into public health policies. ***Level of Evidence II; Therapeutic Studies— Investigating the Results of Treatment***.

## RESUMO

**Objetivo:**

Considerando a necessidade de assistência especializada para Osteogênese Imperfeita (OI), investigamos se o uso de uma plataforma digital pode contribuir para otimizar o tratamento e melhorar a qualidade de vida dos pacientes.

**Métodos:**

Foi desenvolvido um aplicativo de orientação e coleta de dados, complementado por um canal no YouTube com instruções fisioterapêuticas. O ensaio clínico intervencional sem grupo controle foi realizado por 12 meses com pacientes portadores de OI. A funcionalidade dos pacientes foi avaliada utilizando a escala Medida de Independência Funcional (MIF). Os pacientes foram acompanhados por telemedicina, recebendo suporte multidisciplinar remoto.

**Resultados:**

Treze participantes concluíram o estudo. Houve melhoria significativa nos subitens de autocuidados, transferências e locomoção (p<0,05). A aderência ao programa foi elevada e nenhum paciente sofreu lesão durante a intervenção. Além disso, foi observada uma economia total com transporte de R$ 113.852,00, reduzindo os custos diretos para os pacientes e suas famílias.

**Conclusão:**

A telemedicina foi eficaz na reabilitação de pacientes com OI, proporcionando benefícios clínicos e financeiros. A plataforma digital facilitou a adesão ao tratamento, otimizando o suporte médico e fisioterapêutico. Estudos futuros devem explorar sua aplicação em maior escala e sua integração com políticas públicas de saúde. ***Nível de Evidência II; Estudo terapêutico - Investigação dos resultados do tratamento***.

## INTRODUCTION

Telemedicine is defined as the use of digital communication technologies for the provision of remote health care services, allowing remote access to medical care and optimizing the treatment of various clinical conditions.^1^ With the technological evolution and the increasing digitization of health care, this model has been widely adopted to broaden the reach of medical care.^1^

The advancement of telemedicine was especially driven by the COVID-19 pandemic, making it an essential tool to ensure continuity of care in various specialties. However, its potential has been being exploited for decades. One of the first records of its use occurred in 1966, when the Nebraska Psychiatric Institute, in partnership with the Norfolk State Hospital, implemented a telemedicine system for communication, education and psychiatric research using closed circuit televisions.^2,3^

Recent studies demonstrate the effectiveness of telemedicine in different clinical contexts. Shan and collaborators^4^ conducted a systematic review showing that remotely monitored diabetic patients showed better glucose control compared to those treated in person. In addition, 66% of the world's population already owned a smartphone, which expands the possibilities of adherence to this modality of care.^4^

Given the success of telemedicine in the management of chronic diseases, there is a possibility of its application in the field of rare diseases, such as Imperfect Osteogenesis (IO). OI is a genetic disorder characterized by bone fragility, resulting from mutations in the genes COL1A1 and COL1A2, responsible for the synthesis of collagen type I. The prevalence of the disease is estimated at approximately 1 in every 20,000 births in the United States, although specific data on the Brazilian population are not yet available.^5^

IO management requires ongoing multidisciplinary follow-up and specialized care, often unavailable in conventional health centers. In addition, the daily care burden imposed on patients and caregivers is significant. Pinto and colleagues have demonstrated that individuals with rare diseases, including IO, demand about 13 hours a day for essential care only, and moving to health care is one of the main factors that impacts time and family costs.^6^

In view of this scenario, national and international guidelines emphasize the need for innovative strategies for the care of patients with IO.^7^ Telemedicine, in this context, can be an effective alternative to reduce geographical and financial barriers, as well as broaden access to specialist medical support. To evaluate this possibility, we developed an application for guidance and data collection, associated with telemedicine care, with teleconsultations, teleorientation and tele-education. The main objective of this study was to evaluate the functionality of this platform in the follow-up of patients with IO and analyze its impact on treatment costs.

## METHODS

### Development of educational material and application for guidance and data collection

**Figure 1:**
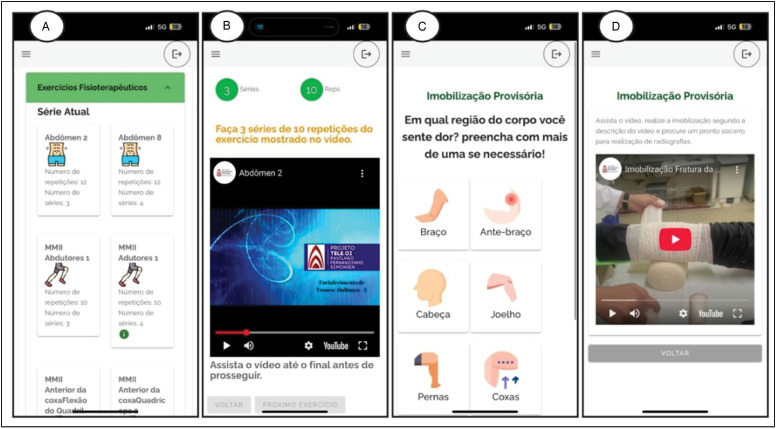
Images that represent the instructions found in the app. A and B: practical examples and videos of recommended physiotherapeutic exercises. C and D: practical examples and videos of provisional immobilization.

Source: Own app developed by the group. (www.orthotech.app).

To enable the conduct of the study, a data collection and telemedicine tool was developed, consisting of a separate application and a YouTube channel with visual guidance on strengthening exercises and immobilization techniques for fractures (Figure 1). The platform was developed using the JavaScript programming language and works both as an environment for data collection and as a virtual medium to offer physiotherapeutic guidance and pre-hospital care, in addition to allowing the reporting of emergency situations. The YouTube channel – *Ortopedia Pediátrica Santa Casa* ^8^ – was created to assist patients at home, providing instructive videos on daily strength exercises (Fig. 1A and 1B) and pre-hospital immobilization techniques in cases of fractures (Fig. 1C and 1D). These videos are also integrated into the app through an automated system based on "Decision Trees", allowing direct access without the need to connect to YouTube. Access to the platform takes place through the domain www.orthotech.app, restricted to users authorized by the controller.

All patients underwent a first face-to-face consultation and subsequent telemedicine treatments were carried out within the outpatients of the Department of Orthopedics of the Santa Casa de São Paulo, using the electronic medical records.

In addition, an algorithm was developed to estimate the resource savings per physical therapy session, based on the *Treatment Outside the Home* (TOH)^9^ and the *Atende* Program of the Prefecture of São Paulo.^10^ The calculation took into account the distance between the patient's residence and the Santa Casa de São Paulo, classifying patients according to the applicable transport program. For residents in the capital, the cost was estimated based on the *Atende* Program, while for those outside, the TOH was used. Considering the average price of gasoline at R$ 5.00 during the intervention period, the app automatically calculated the cost of transportation per physical therapy session according to the record of the exercises on the platform.

## CLINIC TESTING

Following the development of the digital tools, an interventional clinical trial, without a control group (Level of Evidence III), was conducted in the period from October 2022 to October 2023, with patients with Imperfect Osteogenesis (IO) treated in the Reference Center in Imperfect Osteogenesis (CROI) of the Orthopedics and Traumatology Service of Santa Casa de São Paulo. The inclusion criteria covered patients over three years of age, who had IO, who had a smartphone with Internet access and the ability to support the Google Meet program, used in teleconsultations. Patients under three years old, those without any kind of internet access, individuals on pamidronate treatment and elderly patients who, due to lack of familiarity with mobile devices, could not use the app or follow the therapy remotely were excluded. The Sillence classification^11^ was not considered an inclusion or exclusion criterion.

During the study, patients received monthly multidisciplinary follow-up, conducted digitally and synchronously (online), with an orthopedist and a physiotherapist. The frequency of follow-up was changed only in cases of fracture. During the intervention period of one-year, digital physiotherapy was prescribed, recommending the activities to be performed at least three times a week, asynchronously (offline). The multidisciplinary telemedicine consultations were held every three months, when the physiotherapeutic training was adjusted to optimize muscle stimulation. For younger patients, it was recommended to use light bullets or toys as substitutes for the elastics. In addition, plastic pools were provided for walking training in a water environment in the cases of deformities of the lower limbs, as described in the literature.^12^ In the initial consultation, the degree of functional independence of patients was evaluated through the Functional Independence Measurement (FIM), an instrument previously translated and validated into Brazilian Portuguese.^13^

### Statistical analysis

For each FIM subitem, the data was submitted to Student's t test for paired samples. The adopted level of significance was of *p*<0.05.

### Ethical aspects

The project was approved by the Research Ethics Committee of the Brotherhood of Santa Casa de Misericórdia de São Paulo under the number CAAE 42783021.3.0000.5479 and the clinical trial was registered in the *Clinical Trials* (NCT06555536).

## RESULTS

The study included 15 participants, 7 women and 8 men, aged between 3 and 47 years, followed for a period of 12 months. Two participants quit the study, resulting in a total of 13 patients who completed the intervention. According to Sillence classification,^12^ 1 patient (7.7%) had Imperfect Osteogenesis Type I, 6 (46.1%) were classified as Type III and 6 (46.1%) as Type IV. Among the patients who discontinued participation, a 15-year-old female teenager chose to discontinue the intervention, while a 47-year-old male patient reported difficulties in using the app.

**Figure 2:**
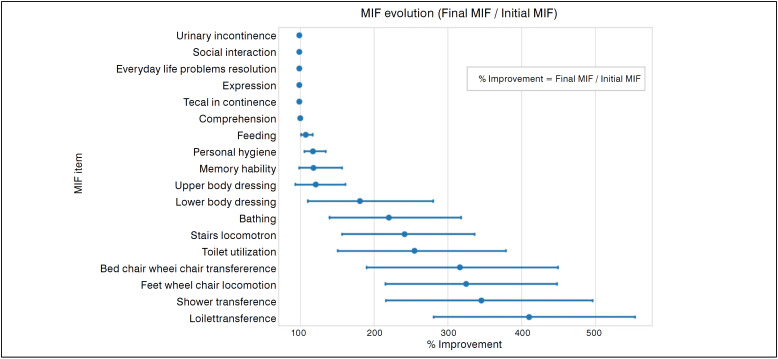
Hypothesis test for each FIM item (Ho: FIM before = FIM after). The circles represent the values of p and the color indicates the statistical significance (p<0.05). Values of p less than 0.05, the null hypothesis at the 95% confidence level is rejected, assuming there was an improvement in the FIM.

**Figure 3:**
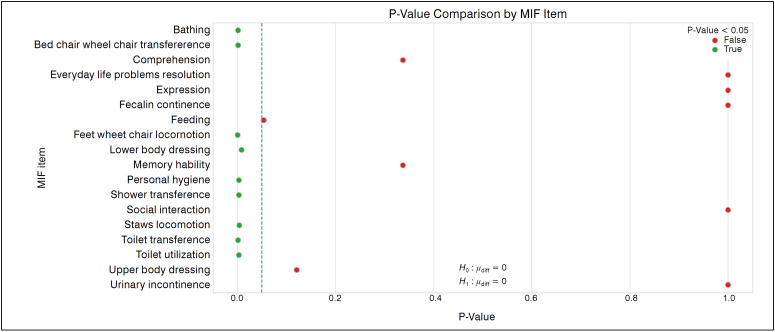
Percentage variation of FIM items, calculated by the ratio (final FIM/initial FIM) x 100. Values of 100% indicate no improvement, while 300% triple the initial value.

The functional evaluation was carried out through the Functional Independence Measure (FIM), applied at the start of the intervention, after three months and at the end of the follow-up. To optimize the analysis, an automated algorithm was developed based on the document "Functional Guidance for the Use of FIM",^14^ aimed at standardizing the application of the scale and minimizing intra and inter observers variations. The FIM is subdivided into 18 subpoints, including food, personal hygiene, bathing, clothing, toilet use, sphincter control, transfers, mobility, communication, social interaction, problem solving and memory. The intervention demonstrated statistical significance (*p*<0.05) in subpoints related to self-care, transfers and locomotion (Figure 2). The largest individual gains in FIM were observed in transfers to toilet (up to 550%), shower (up to 500%) and from bed to wheelchair (up to 450%). (Figure 3)

No patient suffered injuries related to the practice of distance-oriented physiotherapeutic exercises. Adherence to the program was high, with 13 of the 15 participants using the app at least three times a week throughout the year of intervention.

**Figure 4:**
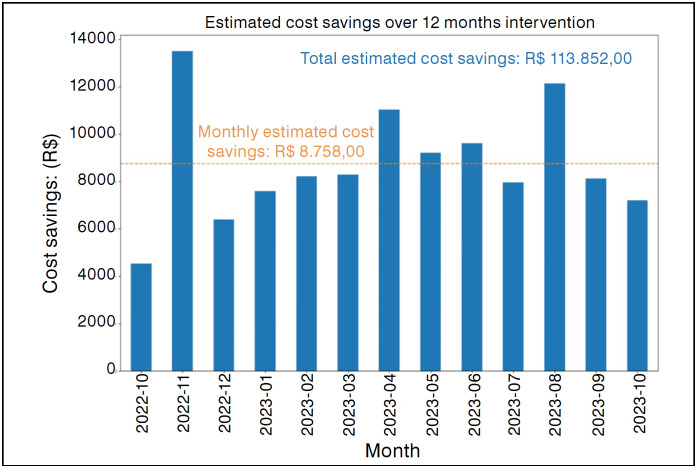
Economy (in real) generated by intervention activities over twelve months.

At the end of the study, the total cost saved with transportation was R$ 113,852.00, corresponding to a monthly average of R$ 8,758.00 per patient over the 12 months of follow-up. (Figure 4)

## DISCUSSION

Our results showed that the use of telemedicine in the treatment of Imperfect Osteogenesis (IOS) resulted in significant functional gains, especially in the items of the Functional Independence Measure (FIM) related to transfers (toilet, shower and from the wheelchair bed). In addition, we observed substantial savings with transport over the intervention period.

One of the challenges frequently mentioned in the literature on IO and other rare diseases is the impact of continuous care on caregivers, both in terms of dedicated time and financial impact on family income. Studies indicate that time spent in care can generate indirect costs due to the reduction in working hours or even the caregiver's exit from the professional market.^6^ In our intervention, caregivers report not only functional improvements in patients (such as greater independence to dress and make transfers), but also an increase in time and quality of life for themselves.

**Figure 5:**
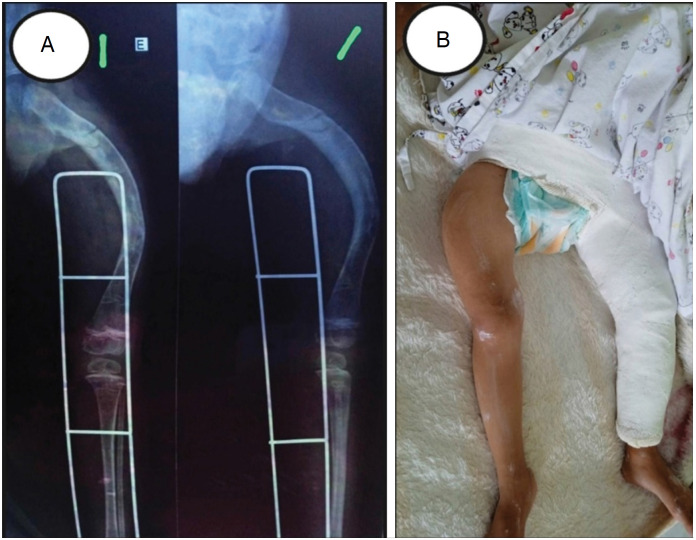
X-ray image of left femur fracture (A) and immobilized left lower limb photograph of patient with fracture (B).

Source: Author.

Another relevant aspect was the possibility of following patients inside their homes through telemedicine. This approach has identified specific challenges of the household environment, including precarious housing conditions that can contribute to the increased incidence of fractures. From these observations, it was possible to suggest customized adaptive strategies for each case. In addition, telemedicine enabled the implementation of a teleorientation service for caregivers and health professionals in municipalities in the interior of São Paulo and Minas Gerais. This initiative included from instructions for pre-hospital care to guidelines for proper immobilization of fractures. In one case, an orthopedic immobilization technician was remotely guided on the best technique for stabilization, allowing pain control until the patient was transferred to a specialized service (Figure 5). Similarly, we were able to assist a generalist orthopedist in the therapeutic decision, contraindicating the use of plates and screws – an inappropriate approach for patients with IO, as it can result in unfavorable outcomes.^15^

From a physiotherapeutic point of view, the use of the FIM Scale allowed not only to quantify the individual limitations of patients, but also to adapt the training in a personalized way. At the end of the intervention period, the re-evaluation with the FIM demonstrated the effectiveness of the applied approach. Adherence to the program was high, and no patient suffered injuries due to remote exercises. We believe that implementation of engagement strategies, such as application gamification, can further improve participant adherence.

One limitation of the study was the absence of a control group. Due to the rarity of the disease and the refusal of the patients to participate if there was a possibility of allocation in the control group, we chose to offer the intervention to all 15 participants.

In general, rare diseases require innovative solutions to ensure effective therapies, as the diagnosis and treatment of these patients still presents significant challenges. It is estimated that about 50% of individuals with suspected rare disease remain unconfirmed.^16^ In Europe, approximately 25% of these patients take between 5 and 30 years to get a definitive diagnosis, and 40% undergo incorrect diagnoses and inadequate treatments.^17^ In Brazil, these data are still not well documented.

As far as medication is concerned, the situation is even more critical: less than 3% of rare diseases have a specific pharmacological therapy, and the costs of these medications can be up to 13.8 times higher than those of conventional drugs.^18,19^ In response to this problem, the *International Rare Diseases Research Consortium* (IRDiRC) was created in 2011 with the aim of fostering international collaboration for the development of new diagnostic and treatment methodologies. In 2017, this organization established a 10-year strategic plan, with one pillar focused on assessing the impact of treatments for rare diseases.^20^ We hope our findings can contribute to these guidelines and encourage new approaches to the management of patients with IO.

Finally, the developed digital tool proved to be effective both in expanding the access to specialist telemedicine care and in improving the function of patients, in addition to providing significant savings with transportation for face-to-face consultations.

## CONCLUSION

Our results indicate that the approach applied in this study may be an important ally in the treatment of Imperfect Osteogenesis, contributing to the improvement of the quality of life of patients and reducing the costs associated with treatment. Although the number of participants has been limited, this restriction is justified by the rarity of the disease.

Similar digital tools should be considered for public health policies, expanding access to specialized therapies for patients with rare diseases. Future research with larger samples will be fundamental to more comprehensively assess the clinical outcomes and the impact of this approach in the long run.

## Data Availability

The data will be made available when requested.
